# Prognostic impact of CDKN2A/B deletion, TERT mutation, and EGFR amplification on histological and molecular IDH-wildtype glioblastoma

**DOI:** 10.1093/noajnl/vdaa126

**Published:** 2020-09-18

**Authors:** Sirui Ma, Soumon Rudra, Jian L Campian, Sonika Dahiya, Gavin P Dunn, Tanner Johanns, Michael Goldstein, Albert H Kim, Jiayi Huang

**Affiliations:** 1 Department of Radiation Oncology, Washington University School of Medicine, St. Louis, Missouri, USA; 2 Department of Medicine, Oncology Division, Washington University School of Medicine, St. Louis, Missouri, USA; 3 Department of Pathology and Immunology, Washington University School of Medicine, St. Louis, Missouri, USA; 4 Department of Neurological Surgery, Washington University School of Medicine, St. Louis, Missouri, USA

**Keywords:** CDKN2A/B, cIMPACT-NOW, EGFR, Glioblastoma, TERT

## Abstract

**Background:**

We aimed to evaluate the clinical outcomes of molecular glioblastoma (mGBM) as compared to histological GBM (hGBM) and to determine the prognostic impact of *TERT* mutation, *EGFR* amplification, and *CDKN2A/B* deletion on isocitrate dehydrogenase (IDH)-wildtype GBM.

**Methods:**

IDH-wildtype GBM patients treated with radiation therapy (RT) between 2012 and 2019 were retrospectively analyzed. mGBM was defined as grade II-III IDH-wildtype astrocytoma without histological features of GBM but with one of the following molecular alterations: *TERT* mutation, *EGFR* amplification, or combination of whole chromosome 7 gain and whole chromosome 10 loss. Overall survival (OS) and progression-free survival (PFS) were calculated from RT and analyzed using the Kaplan–Meier method. Multivariable analysis (MVA) was performed using Cox regression to identify independent predictors of OS and PFS.

**Results:**

Of the 367 eligible patients, the median follow-up was 11.7 months. mGBM and hGBM did not have significantly different OS (median: 16.6 vs 13.5 months, respectively, *P* = .16), nor PFS (median: 11.7 vs 7.3 months, respectively, *P* = .08). However, mGBM was associated with better OS (hazard ratio [HR] 0.50, 95% CI 0.29–0.88) and PFS (HR 0.43, 95% CI 0.26–0.72) than hGBM after adjusting for known prognostic factors on MVA. *CDKN2A/B* deletion was associated with worse OS (HR 1.57, 95% CI 1.003–2.46) and PFS (HR 1.57, 95% CI 1.04–2.36) on MVA, but *TERT* mutation and *EGFR* amplification were not.

**Conclusion:**

Criteria for mGBM may require further refinement and validation. *CDKN2A/B* deletion, but not *TERT* mutation or *EGFR* amplification, may be an independent prognostic biomarker for IDH-wildtype GBM patients.

**Key Points**

- Molecularly defined GBM based on the cIMPACT-NOW criteria has more favorable prognosis than histological GBM.
- *CDKN2A/B* deletion predicts worse survival for IDH-wildtype GBM.
- *TERT* and *EGFR* alterations are not prognostic markers for IDH-wildtype GBM.

**Importance of the Study**

Molecular and genomic-profiling techniques have improved our understanding of glioblastoma (GBM). Common genetic alterations in GBM include *TERT* mutation, *EGFR* amplification, and *CDKN2A/B* deletion, but their clinical impact remains unclear. A recently proposed consensus statement from the cIMPACT-NOW committee has defined a new diagnostic entity termed as “diffuse astrocytic glioma, IDH-wildtype with molecular features of GBM, WHO grade IV.” We performed this large institutional study to evaluate the prognostic impact of these common molecular alterations and this molecularly defined GBM entity among IDH-wildtype GBM. Our results demonstrated that the molecularly defined GBM had better survival compared with histologic GBM when adjusted for other clinical or treatment factors. Moreover, we demonstrated that homozygous *CDKN2A/B* deletion was an independently prognostic biomarker for worse survival among IDH-wildtype GBM, whereas *TERT* mutation and *EGFR* amplification were not. Our data support additional clinical investigation to validate CDKN2A/B deletion as a prognostic biomarker for IDH-wildtype GBM.

Glioblastoma (GBM) is the most common malignant primary brain tumor with an exceedingly poor prognosis despite multimodality treatment.^1,2^ It represents a heterogeneous entity with an expansive molecular and mutational landscape.^3^ The recently updated World Health Organization (WHO) classifications have now incorporated some of the new molecular advances in their categorization and notably distinguishes GBM by isocitrate dehydrogenase (IDH) mutation status.^4^ Significant advances in molecular and genetic techniques have allowed for the detailed analysis of genomic alterations in GBM. These efforts have yielded an emerging understanding of the dysregulating alterations in 3 key molecular signaling pathways, namely receptor tyrosine kinase/phosphoinositide 3-kinase (RTK/PI3K), p53, and Rb, as obligatory events in GBM tumorigenesis.^5,6^ Further gene expression–based molecular studies have facilitated tumor classification into clinically relevant subtypes that may exhibit distinct treatment response characteristics.^7^

Additional insight into somatic mutation profiles and their impact on tumor behavior is necessary for diagnostic clarity, prognostication, and identifying potential therapeutic targets. However, there is uncertainty regarding the prognostic value of some of the most common genetic alterations in GBM, such as telomerase reverse transcriptase (*TERT)* promoter mutation, epidermal growth factor receptor (*EGFR*) amplification, and cyclin-dependent kinase inhibitor 2A/B (*CDKN2A/B*) deletion.^8,9^  *CDKN2A/B* deletion appears prognostic for IDH-mutant astrocytoma,^10,11^ but its impact on the clinical outcomes of IDH-wildtype GBM has not been extensively investigated. The Consortium to Inform Molecular and Practical Approaches to CNS Tumor Taxonomy (cIMPACT-NOW) recently proposed a diagnostic entity of grade II-III IDH-wildtype astrocytoma that should behave similarly as histological GBM (hGBM): *diffuse astrocytic glioma, IDH-wildtype with molecular features of glioblastoma, WHO grade IV* (referred hereafter as molecular GBM). The molecular features defining this new tumor entity include *TERT* mutation, *EGFR* amplification, or a combination of whole chromosome 7 gain and whole chromosome 10 loss.^12^ However, because grade II-III astrocytomas were not uniformly treated as aggressively as GBM in the past, additional clinical data should validate that molecular GBM (mGBM) has comparable clinical outcomes to hGBM after modern chemoradiotherapy.

To address the above questions, this retrospective study aims to leverage our large institutional data to evaluate the clinical outcomes of mGBM as compared to hGBM and to elucidate the prognostic impact of *TERT* mutation, *EGFR* amplification, and *CDKN2A/B* deletion on IDH-wildtype GBM.

## Methods

### Patient Population

Adult patients aged 18 years and older with newly diagnosed WHO grade IV IDH-wildtype supratentorial hGBM or mGBM who were treated with at least one fraction of radiation therapy (RT) with or without chemotherapy at our tertiary cancer center from July 2012 to July 2019 were retrospectively reviewed. mGBM was defined as histological grade II-III *IDH*-wildtype astrocytoma with at least 1 of the following molecular alterations: *TERT* promoter mutation, *EGFR* amplification, or a combination of whole chromosome 7 gain and whole chromosome 10 loss in concordance with a recently published recommended diagnostic criteria.^12^ Patients were required to have known *O*^6^-methylguanine–DNA methyltransferase (*MGMT*) promoter methylation status. Exclusion criteria included known IDH mutation, *H3 K27M* mutation, leptomeningeal disease, gliomatosis, or infratentorial disease in the brainstem or cerebellum. The start date from July 2012 was chosen for analysis as that was when routine testing of *MGMT* and IDH become our institutional practice. The study was conducted with the approval of the institutional review board.

### Pathologic and Molecular Analysis

All tumor specimens were evaluated by the institutional board–certified neuropathologists. IDH mutation status was assessed by immunohistochemistry to detect IDH1-R132H mutation or next-generation sequencing (NGS) to assess for variants in *IDH1* or *IDH2* genes. *EGFR* amplification was identified via fluorescence in situ hybridization (FISH) and/or NGS. EGFR amplification on FISH was defined if the EGFR probe to chromosome 7 probe ratio ≥ 2.0^13,14^ and was considered the gold standard if yielded the discrepant result as compared to NGS.^15^  *TERT* mutation and homozygous *CDKN2A/B* deletion were only evaluated by NGS. NGS was mostly performed using the commercial Foundation one CDx test (F1CDx, Foundation Medicine), which is a Clinical Laboratory Improvement Amendments (CLIA)-certified NGS diagnostic test to detect substitutions, insertions, deletions, and copy number alterations in 324 genes and select gene rearrangements using DNA isolated from formalin-fixed paraffin-embedded (FFPE) tumor tissue specimens. It is an FDA-approved tissue-based companion diagnostic test for tumor mutation profiling to be used by qualified health care professionals (https://www.accessdata.fda.gov/cdrh_docs/pdf17/P170019C.pdf). Some patients had a CLIA-certified institutional NGS panel called GPS test (https://gps.wustl.edu/wp-content/uploads/2015/11/CNS_tumor_info_card_UTD.pdf) that used targeted hybridization capture coupled with NGS of tumor-derived genomic DNA from FFPE tissues to evaluate 24 genes commonly involved in CNS tumors, including *IDH1/2* and *TERT* mutations, but it did not evaluate *EGFR* amplification nor *CDKN2A/B* loss. *TERT* mutation, *EGFR* amplification, and *CDKN2A/B* deletion status were obtained based on the analysis of the tumor samples at the initial diagnosis.

### Treatments

Extent of resection (EOR) was classified into the following 3 categories: biopsy, subtotal resection (STR), and gross-total/near-total resection (GTR) based on operative report and postoperative MRI as previously described.^16^ Patients received either standard-course RT (SRT) over approximately 6 weeks or short-course hypofractionated RT (HFRT) as previously described.^17,18^ Patients treated with dose-escalation protocols with a simultaneous boost with fractional dose > 2 Gy/day to a subregion as part of a 6-week RT course, such as on the NRG-BN001 study (NCT02179086), were included in the SRT cohort, as were patients who started on SRT but did not complete or switched to HFRT. Concurrent TMZ at a dose of 75 mg/m^2^ was given daily during RT. Adjuvant TMZ was typically initiated 4–6 weeks after completion of RT and administered orally at a dose of 150–200 mg/m^2^ given on days 1–5 per 28-day cycle for 6–12 cycles at the discretion of the neuro-oncologists.

### Statistics

Patient and treatment characteristics were compared using Fisher’s exact test for categorical variables and Mann–Whitney *U* test for continuous variables. Overall survival (OS) and progression-free survival (PFS) were calculated using the Kaplan–Meier method and compared using the log-rank test. All time-to-event data were calculated from the start of RT. Univariable analysis (UVA) and multivariable analysis (MVA) were performed using the Cox proportional hazards regression model to identify prognostic factors associated with survival outcomes. Proportional hazard assumptions of each variable were checked graphically by using a log-log survival plot. Variables with *P* less than .20 on UVA and well-established prognostic factors for survival were entered into the MVA. For the biomarkers that were not available for the entire cohort, they were not entered for the MVA of the entire cohort but were analyzed separately for the subset analyses. All statistical tests were 2 sided. Statistical analyses were performed with the Statistical Package for Social Sciences, version 23.0 (IBM SPSS Statistics).

## Results

### Patient Characteristics

Among 466 patients screened during the study period, a total of 367 adult patients with newly diagnosed supratentorial/nonmetastatic histological GBM (hGBM) or molecular GBM (mGBM) met the eligibility criteria and were included in this study. The following patients were excluded from analysis: 57 patients with unknown *MGMT* status, 26 patients with IDH-mutant GBM, 12 patients with leptomeningeal disease or gliomatosis, 3 infratentorial GBM, and 1 *K27M*-mutant diffuse midline glioma. Table 1 lists patient and treatment characteristics for the entire cohort and separately for the hGBM and mGBM subsets. The median age was 60, and the median KPS was 80. The majority of patients had GTR (50%), harbored unmethylated *MGMT* (63%), treated with SRT (84%), and received TMZ (89%). The median SRT dose was 60 Gy, and the median HFRT dose was 40 Gy. Of the 328 patients who received TMZ, 327 (99.9%) received concurrent TMZ as per the Stupp protocol, and only 1 case received adjuvant TMZ after RT alone because he was on a protocol that omitted concurrent TMZ. Overall, 350 of them (95%) were confirmed to be IDH-wildtype on immunohistochemistry or NGS, and the remaining 17 cases were all hGBM who had no known IDH mutation but also lacked information on *TERT* mutation, *EGFR* amplification, and *CDKN2A/B* deletion status. NGS was performed on tumor specimens from 150 patients using F1CDx and on additional 46 cases using GPS. Overall, *TERT* mutation was evaluated using NGS for 184 cases (150 using F1CDx and 34 using GPS); *EGFR* amplification was evaluated for 277 cases (22 with FISH alone, 150 with F1CDx NGS alone, and 105 with both FISH and NGS); and *CDKN2A/B* deletion was evaluated for 150 cases using F1CDx NGS. Of the 105 patients who had evaluation of *EGFR* amplification by both FISH and NGS, 98% had identical results, with only 2 cases that were positive on FISH but not on NGS (both are counted as positive for the analysis). Of the 93 cases with *CDKN2A/B* deletion, 90 had deletions of both *CDKN2A* and *CDKN2B*, while 3 had *CDKN2A* deletion only. Twenty-two patients (6%) had mGBM (7 patients with grade II and 15 patients with grade III astrocytoma based on the 2016 WHO grading criteria). There were 26 other supratentorial/nonmetastatic IDH*-*wildtype grade II–III astrocytoma cases during the study period: 12 were evaluated but did not have any of the molecular alterations of mGBM, and the remaining 14 cases were not evaluated.

**Table 1.** Patient and Treatment Characteristics

Characteristics	All (*n* = 367)	hGBM (*n* = 345)	mGBM (*n* = 22)	*P* Value
GBM classification, *n* (%)		—	—	—
hGBM	345 (94)			
mGBM	22 (6)			
Age at diagnosis (y), median (range)	60 (21–86)	60 (21–86)	62.5 (37–80)	.97
KPS, median (range)	80 (30–100)	80 (40–100)	90 (30–100)	.06
Sex, *n* (%)				
Male	219 (60)	207 (60)	12 (55)	.66
Female	148 (40)	138 (40)	10 (45)	
Race, *n* (%)				
White	344 (94)	324 (94)	20 (91)	.64
Other	23 (6)	21 (6)	2 (9)	
Extent of resection, *n* (%)				
GTR	182 (50)	177 (51)	5 (23)	<.01
STR	99 (27)	95 (28)	4 (18)	
Biopsy	86 (23)	73 (21)	13 (59)	
*MGMT* methylation, *n* (%)				
Yes	135 (37)	128 (37)	7 (32)	.82
No	232 (63)	217 (63)	15 (68)	
RT type, *n* (%)				
SRT	307 (84)	287 (83)	20 (91)	.55
HFRT	60 (16)	58 (17)	2 (9)	
TMZ chemotherapy				
Yes	328 (89)	308 (89)	20 (91)	1.00
No	39 (11)	37 (11)	2 (9)	
*TERT* mutation by NGS, *n* (%)				
Yes	167 (91)	151 (90)	16 (94)	1.00
No	17 (9)	16 (10)	1 (6)	
Unknown	183			
*EGFR* amplification by FISH or NGS				
Yes	118 (43)	112 (44)	6 (29)	.25
No	159 (57)	144 (56)	15 (71)	
Unknown	90			
*CDKN2A/B* deletion by NGS				
Yes	93 (62)	87 (64)	6 (40)	.09
No	57 (38)	48 (36)	9 (60)	
Unknown	217			
Somatic mutations of the 3 canonical pathways^a^				
Three pathways	130 (87)	119 (88)	11 (73)	.23
Two pathways	13 (9)	10 (7)	3 (20)	
One pathway	7 (5)	6 (4)	1 (7)	
Unknown	217			

FISH, fluorescence in situ hybridization; GTR, gross-total/near-total resection; HFRT, short-course hypofractionated RT; hGBM, histological GBM; mGBM, molecular glioblastoma; NGS, next-generation sequencing; RT, radiation therapy; SRT, standard-course RT; STR, subtotal resection; TMZ, temozolomide.

^a^Somatic mutations affecting PI3K/MAPK, p53, or Rb pathways as detected on a commercial NGS of 324 gene panel.

### Comparison of Molecular and Histological GBM

**Figure 1.**
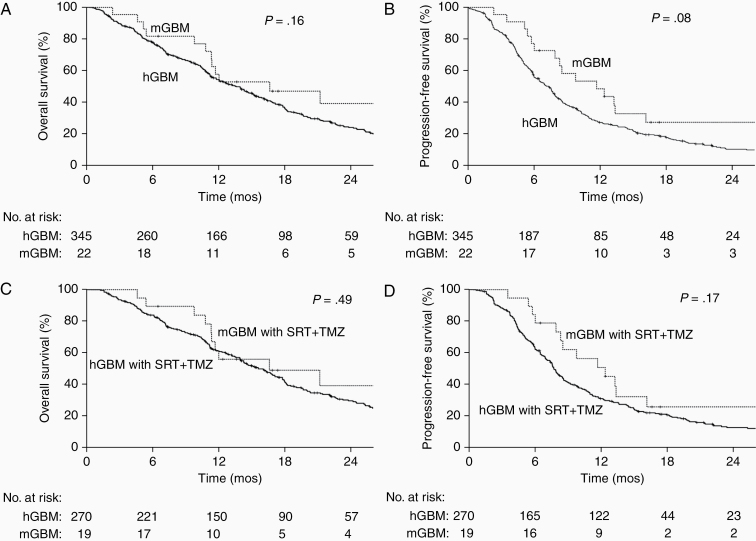
Survival outcomes of patients stratified by molecular glioblastoma (mGBM) and histological GBM (hGBM) status. (A) Overall survival (OS) of the entire study cohort. (B) Progression-free survival (PFS) of the entire study cohort. (C) OS of the subset receiving standard-course radiation therapy (SRT) and temozolomide (TMZ). (D) PFS of the subset receiving SRT and TMZ.

After a median follow-up of 11.7 months, the median OS and PFS for the entire cohort were 13.9 months (95% CI 12.2–15.6) and 7.5 months (95% CI 6.8–8.3), respectively. Patients with mGBM had nonsignificantly higher OS (median: 16.6 vs 13.5 months, respectively, *P* = .16; Figure 1A) and PFS (median: 11.7 vs 7.3 months, respectively, *P* = .08; Figure 1B) as compared to patients with hGBM. When evaluating a more homogeneous subset of patients who received SRT and TMZ, similar trend was observed between mGBM and hGBM for OS (median: 16.6 vs 15.6 months, respectively, *P* = .49; Figure 1C) and PFS (median: 12.4 vs 7.7 months, respectively, *P* = .17; Figure 1D). The reason that the survival curves of mGBM and hGBM after SRT+TMZ appeared closer was likely due to selection bias in clinical practice: More clinically aggressive mGBM (higher grade or larger tumor burden IDH-wildtype astrocytomas) were historically treated with standard chemoradiotherapy, whereas better prognostic hGBM (younger patients with better KPS) received standard chemoradiotherapy. As given in Table 1, the mGBM cohort had a lower proportion of GTR, a nonsignificantly higher KPS, and a nonsignificantly lower proportion of CDKN2A/B deletion when compared with the hGBM cohort. On MVA, mGBM was associated with improved OS (hazard ratio [HR] 0.50, 95% CI 0.29–0.88) and PFS (HR 0.43, 95% CI 0.26–0.72) relative to hGBM after adjusting for known prognostic factors (Table 2; Supplementary Table S1). Interestingly, age lost its significance for OS and PFS on MVA, likely due to its correlation with the use of HFRT and TMZ, which are the 2 treatment factors included in the multivariable models. Similar results were also obtained using MVA with a more homogenous subset of patients treated with SRT and TMZ (data not shown). Of note, the OS and PFS between mGBM with histological grade II versus grade III astrocytoma were similar (data not shown). One mGBM patient with histological grade II astrocytoma and *TERT* mutation is still alive at 62 months, and he was treated with subtotal resection followed by RT alone without chemotherapy.

**Table 2.** Univariable and Multivariable Analysis for OS

Characteristics	UVA	*P* Value	MVA	*P* Value
Age at diagnosis	1.02 (1.01–1.03)	<.001	1.00 (0.99–1.02)	.39
KPS	0.97 (0.96–0.98)	<.001	0.98 (0.97–0.99)	.001
Male sex	1.03 (0.82–1.21)	.78	—	—
Non-White race	1.07 (0.65–1.77)	.80	—	—
mGBM (vs hGBM)	0.68 (0.40–1.17)	.16	0.50 (0.29–0.88)	.02
Extent of resection				
GTR	Ref			
STR	1.56 (1.18–2.06)	.002	1.66 (1.24–2.22)	.001
Biopsy	3.264 (2.42–4.40)	<.001	3.05 (2.22–4.17)	<.001
Unmethylated *MGMT*	1.79 (1.39–2.30)	<.001	1.81 (1.40–2.35)	<.001
HFRT	2.48 (1.83–3.37)	<.001	1.53 (1.04–2.24)	.03
No TMZ chemotherapy	2.68 (1.86–3.85)	<.001	1.61 (1.07–2.41)	.02
*TERT* mutation (*n* = 184)	1.14 (0.64–2.03)	.67	—	—
*EGFR* amplification by FISH or NGS (*n* = 277)	1.21 (0.92–1.58)	.18	—	—
*CDKN2A/B* deletion by NGS (*n* = 150)	1.46 (0.96–2.21)	.08	—	—
Pathways affected			—	—
Three pathways	Ref			
Two pathways	0.71 (0.33–1.53)	.38		
One pathway	0.32 (0.08–1.28)	.11		

FISH, fluorescence in situ hybridization; GTR, gross-total/near-total resection; HFRT, short-course hypofractionated radiation therapy; hGBM, histological GBM; mGBM, molecular glioblastoma; MVA, multivariable analysis; NGS, next-generation sequencing; STR, subtotal resection; TMZ, temozolomide; UVA, univariable analysis.

### Prognostic Impact of TERT Mutation, EGFR Amplification, and CDKN2A/B Deletion

**Figure 2.**
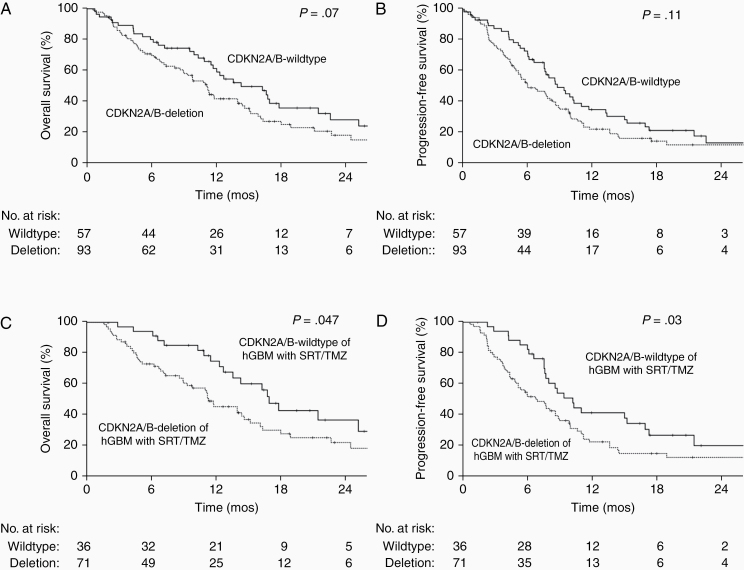
Survival outcomes of patients stratified by CDKN2A/B deletion status. (A) Overall survival (OS) of the entire study cohort. (B) Progression-free survival (PFS) of the entire study cohort. (C) OS of the subset receiving standard-course radiation therapy (SRT) and temozolomide (TMZ). (D) PFS of the subset receiving SRT and TMZ.

As given in Table 2 and Supplementary Table S1, *TERT* mutation, *EGFR* amplification, and *CDKN2A/B* deletion were not significantly associated with different OS, nor for PFS on UVA. Because these 3 biomarkers were not uniformly tested for the entire cohort, each variable was evaluated separately in the subset of patients with available information on their mutation status. On MVA, *CDKN2A/B* deletion was associated with worse OS (HR 1.57, 95% CI 1.003–2.46) and PFS (HR 1.57, 95% CI 1.04–2.36) after adjusting for other known prognostic factors including *MGMT* methylation (Table 3). However, *TERT* mutation and *EGFR* amplification were not significantly associated with different OS, nor PFS on MVAs (Supplementary Tables S2–S3). There was no significant interaction between *MGMT* methylation and *TERT* mutation for OS or PFS (data not shown). To reduce the effect of heterogeneous treatment and potentially more favorable outcomes of mGBM, the MVA was then repeated using a subset of 107 hGBM patients with available *CDKN2A/B* status who received SRT and TMZ. In the subset analysis, *CDKN2A/B* deletion was again associated with significantly worse OS (HR 1.93, 95% CI 1.08–3.43) and PFS (HR 2.02, 95% CI 1.20–3.41; Table 4). As shown in Figure 2A and B, among the entire cohort, *CDKN2A/B* deletion was associated with nonsignificantly worse OS (median: 11.1 vs 14.3 months, respectively, *P* = .07) and PFS (median: 6.0 vs 8.7 months, respectively, *P* = .11) as compared to *CDKN2A/B* wildtype. However, in the more homogenous subset of hGBM patients who received SRT and TMZ, *CDKN2A/B* deletion was associated with statistically worse OS (median: 11.3 vs 16.9 months, respectively, *P* = 0.047; Figure 2C) and PFS (median: 6.9 vs 10.2 months, respectively, *P* = .03; Figure 2D). Interestingly, even in the subset analyses, mGBM was associated with more favorable OS and PFS than hGBM when adjusted for *CDKN2A/B* deletion, *TERT* mutation, or *EGFR* amplification status (Table 3; Supplementary Tables S2–S3). Because 150 patients had a more comprehensive NGS panel of 324 genes using F1CDx, their mutation profiles were reviewed and categorized by alterations in the 3 canonical pathways (RTK/PI3K, p53, and Rb). Interestingly, the majority of patients had somatic mutations in all 3 pathways (87%), with relatively few cases with alterations in only one pathway (5%). The number of pathways involved was not significantly associated with OS, nor PFS on UVA (Table 2; Supplementary Table S1).

**Table 3.** Multivariable Analysis of OS and PFS for All Patients with Known *CDKN2A/B* Deletion Status (*n* = 150)

Characteristics	OS	*P* Value	PFS	*P* Value
Age at diagnosis	1.00 (0.98–1.02)	.90	1.00 (0.98–1.02)	.85
KPS	0.98 (0.96–0.995)	.009	0.98 (0.96–0.99)	.001
mGBM	0.54 (0.25–1.18)	.12	0.46 (0.23–0.92)	.03
Extent of resection				
GTR	Ref			
STR	1.88 (1.08–3.27)	.03	2.23 (1.38–3.61)	.001
Biopsy	3.00 (1.79–5.03)	<.001	3.93 (2.38–6.50)	<.001
Unmethylated *MGMT*	1.82 (1.12–2.96)	.02	2.87 (1.80–4.58)	<.001
HFRT	2.40 (1.23–4.67)	.01	1.39 (0.76–2.56)	.29
No TMZ chemotherapy	1.28 (0.66–2.47)	.46	1.16 (0.63–2.15)	.64
*CDKN2A/B* deletion by NGS	1.57 (1.003–2.46)	.048	1.57 (1.04–2.36)	.03

FISH, fluorescence in situ hybridization; GTR, gross-total/near-total resection; HFRT, short-course hypofractionated radiation therapy; hGBM, histological GBM; mGBM, molecular glioblastoma; NGS, next-generation sequencing; OS, overall survival; PFS, progression-free survival; STR, subtotal resection; TMZ, temozolomide.

**Table 4.** Multivariable Analysis of OS and PFS for hGBM Patients s/p SRT and TMZ with Known *CDKN2A/B* Deletion Status (*n* = 107)

Characteristics	OS	*P* Value	PFS	*P* Value
Age at diagnosis	0.99 (0.97–1.02)	.43	0.99 (0.97–1.01)	.36
KPS	0.96 (0.94–0.99)	.001	0.95 (0.94–0.97)	<.001
Extent of resection				
GTR	Ref		Ref	
STR	1.48 (0.76–2.88)	.25	2.00 (1.14–3.50)	.02
Biopsy	2.79 (1.48–5.25)	.001	3.63 (1.97–6.70)	<.001
Unmethylated *MGMT*	1.59 (0.90–2.83)	.11	2.85 (1.66–4.87)	<.001
*CDKN2A/B* deletion by NGS	1.93 (1.08–3.43)	.03	2.02 (1.20–3.41)	.008

GTR, gross-total/near-total resection; hGBM, histological GBM; mGBM, molecular glioblastoma; NGS, next-generation sequencing; OS, overall survival; PFS, progression-free survival; SRT, standard-course RT; STR, subtotal resection; TMZ, temozolomide.

## Discussion

This study demonstrates that the newly defined entity of mGBM, formally known as “diffuse astrocytic glioma, IDH-wildtype with molecular features of glioblastoma, WHO grade IV,” likely comprises of a small proportion of all IDH-wildtype GBM and may have slightly better clinical outcomes than histological IDH-wildtype GBM when adjusted for known prognostic factors. Although *TERT* promoter mutation and *EGFR* amplification may be common canonical alterations for IDH-wildtype GBM that may guide diagnosis and define mGBM, they do not appear prognostic for established IDH-wildtype GBM cases. In contrast, *CDKN2A/B* deletion appears to be an independent prognostic biomarker for IDH-wildtype GBM.

IDH-wildtype gliomas are known to harbor a worse prognosis than their IDH-mutant counterparts,^19^ with some studies suggesting that most histological grade II-III IDH-wildtype astrocytomas may represent unrecognized GBM when subjected to further molecular and genomic-profiling techniques.^20,21^ These tumors demonstrate heterogeneous clinical behavior, which necessitates the development of additional markers for further stratification.^22^ The cIMPACT-NOW consortium attempted to address this need by proposing guidelines for identifying mGBM based on published literature, suggesting shortened survival for IDH-wildtype grade II-III astrocytomas carrying certain alterations.^12,22–25^ The median OS of 16.6 months exhibited by mGBM in the present report was comparable to that of a previously published cohort of IDH-wildtype grade II-III gliomas with *EGFR* amplification or *TERT* mutation.^22^ Similarly, Reuss et al. previously reported a median OS of 19.4 months for 124 lower grade astrocytoma cases with molecularly integrated GBM diagnosis based on methylation and copy number profiles as well as incorporating information on *TERT* mutation, *EGFR* amplification, chromosome 7p gain and 10q loss, or combined chromosome 10q/13q/14q deletion.^21^ Given the median OS of these patients is only a few months better than hGBM, our study supports that they should be treated aggressively as hGBM. However, after adjusting for known prognostic and treatment factors using MVA, our study also demonstrated that mGBM had significantly better OS and PFS than hGBM. One explanation may be that mGBM cases were diagnosed at a slightly earlier stage than their hGBM counterparts, thus benefiting from lead-time bias. A previous global DNA methylation profiling study of a large cohort of brain tumors revealed that *TERT* mutation was the least specific parameter for GBM compared with *EGFR* amplification or whole chromosome 7 gain and whole chromosome 10 loss, suggesting that *TERT* mutation alone may be an insufficient diagnostic marker of GBM.^26^ Interesting, one of our mGBM patient with *TERT* mutation alone had prolonged OS after RT without chemotherapy. Weller et al. previously analyzed a group of IDH-wildtype grade II–III gliomas with GBM-like copy number changes, primarily whole chromosome 7 gain and whole chromosome 10 loss. This GBM-like group exhibited a median PFS of 1.5 years and OS of 2.4 years, which compared more favorably to hGBM survival data, suggesting that the chromosomal 7 and 10 changes alone may be inadequate to diagnose GBM.^24^ Additionally, a recent report showed that *TERT* mutation conferred significantly different prognosis depending on the presence or absence of whole chromosome 7 gain and whole chromosome 10 loss, suggesting that the presently utilized mGBM criteria may be too heterogeneous.^25^ In contrast, Tesileanu et al. recently compared 71 mGBM patients with 192 hGBM patients and observed similar OS. However, their mGBM cohort had significantly higher proportion of biopsy alone (83% vs 17%, respectively, *P* < .001) and lower proportion of chemoradiotherapy than the hGBM cohort (42% vs 90%, respectively, p<0.001). Thus, the less intense treatment of mGBM may have skewed their OS closer to hGBM. Indeed, after adjusting for treatment imbalance on MVA in their supplemental table, hGBM had borderline worse OS than mGBM (HR 1.58, 95% CI 0.98–2.56; *P* = .06).^27^ Also, their analysis did not evaluate *MGMT* status, which might have further confounded their analysis. Thus, further validation of the cIMPACT-NOW diagnostic guideline with a larger cohort is necessary to assess whether these tumors truly behave comparably to hGBM and whether refinement of these molecular signatures may be required to diagnose mGBM accurately.

Our study demonstrated that homozygous *CDKN2A/B* deletion independently predicted for worse OS and PFS among IDH-wildtype GBM. The prognostic significance of *CDKN2A/B* deletion among IDH-wildtype GBM lacks extensive investigation. The *CDKN2A* gene encodes for the protein p14ARF that serves as a tumor suppressor by stabilizing p53 function and cell cycle control. This locus encodes an additional tumor suppressor protein in p16INK4a that inhibits cyclin D to bind cyclin-dependent kinase 4 and 6, preventing the complex to phosphorylate Rb and to promote G1 to S phase transition.^28,29^ Thus, *CDKN2A* deletion promotes tumorigenesis via 2 of the central somatic mutation pathways implicated in GBM pathogenesis and is typically occurring in the presence of *CDKN2B* deletion. In a genomic analysis of 251 GBMs from The Cancer Genome Atlas (TCGA) Research Network, 58% of p53 pathway disruption occurred via *CDKN2A* deletion, and *CDKN2A* deletion was implicated in over 50% of Rb function impairment.^6^  *CDKN2A* deletion was also associated with high proliferative indices and higher tumor grade.^30,31^ In a previous molecular analysis comparing 2 age- and gender-matched groups of GBM with either long or short period until tumor progression (>24 months vs < 6 months), *CDKN2A* deletion status did not carry prognostic significance, but the study was small with only 21 patients for each group.^32^ Among a study of 105 primary gliomas encompassing grade I–IV astrocytomas, oligoastrocytomas, and oligodendrogliomas, *CDKN2A* deletion was found to be associated with poor survival only in the subgroup of GBM patients older than 50 years.^31^ The authors noted increasing rates of *CDKN2A* deletion from low- to high-grade tumors, suggesting that *CDKN2A* deletion represented a molecular change late in tumor progression rather than in initiation. However, the study was conducted before routine interrogation of IDH status and included both IDH-mutant oligodendrogliomas and astrocytomas along with IDH-wildtype GBM, which might have confounded their analysis. Notably, 2 recent studies reported that the *CDKN2A* deletion was prognostic for IDH-mutant astrocytoma, which will be incorporated in the new grading criteria.^10,11^ Our results suggest that *CDKN2A/B* deletion may also be prognostic for IDH-wildtype GBM and deserves further investigation.

*TERT* promoter mutation was not prognostic for OS and PFS of IDH-wildtype GBM in our study, and we did not observe a significant interaction between *TERT* mutation and *MGMT* methylation. *TERT* mutation is typically observed in 70%–80% of GBM genomes and may represent a mechanism by which these tumors perform telomere elongation to achieve limitless replicative potential.^33,34^ Despite an emerging body of evidence characterizing the functional role of *TERT* mutation and its possible clinical utility as a therapeutic target,^33^ there lacks an established consensus on its prognostic value for IDH-wildtype GBM.^34–36^ Furthermore, multiple retrospective studies have suggested that the clinical significance of the *TERT* mutation may rely on the tumor genetic background, particularly that of *MGMT* methylation status.^37–39^ Arita et al. analyzed 452 IDH-wildtype GBM patients (including 58% *TERT*-mutant) and reported that *TERT* mutation was prognostic for OS and PFS. They also observed significant interaction between *TERT* mutation and *MGMT* methylation, in which the clinical outcomes of *TERT*-mutant versus *TERT*-wildtype differed depending on the *MGMT* methylation status.^38^ In contrast, Nguyen et al. analyzed 303 IDH-wildtype GBM (including 75% *TERT*-mutant) and did not observe significant association of *TERT* mutation with OS and PFS. They also observed significant interaction between *TERT* mutation and *MGMT* methylation.^37^ Pekmezci et al. analyzed 309 IDH-wildtype GBM case (including 77% *TERT*-mutant) and reported *TERT* mutation was not associated with OS, but they did not account for *MGMT* status.^39^ Therefore, our study is consistent with the majority of prior studies suggesting that *TERT* mutation is not an independent prognostic factor for IDH-wildtype GBM. Our study did not observe a significant interaction between *TERT* mutation and *MGMT* methylation, which might have been limited by the smaller sample size and only 9% of our evaluable cohort being *TERT*-wildtype.

Similarly, *EGFR* amplification was also not a significant prognostic factor in this analysis. *EGFR* amplification is one of the most common genetic aberrations in GBM, which has garnered this gene locus significant attention for both a possible molecular marker for tumor outcomes and a potential target for treatment. *EGFR* amplification occurred in 44% of our cohort, which is consistent with previous genomic analyses reporting that approximately 50% of GBMs harbored *EGFR* alterations.^6^  *EGFR* is a receptor tyrosine kinase that induces downstream signaling through RTK/PI3K pathways, among others, to induce cellular differentiation and proliferation and play a causal role in tumorigenesis.^40^ Though hypothesized to serve as a potential prognostic biomarker for GBM, there lacks a consensus regarding the impact of *EGFR* amplification on clinical outcomes. Two recent meta-analyses of available *EGFR* outcomes literature have yielded different results.^41,42^ In the meta-analysis by Chen et al., pooling data from 3 GBM studies and 3 anaplastic astrocytoma studies from before 2010, they found no significant difference in OS between those with positive or negative *EGFR* amplification.^41^ In the second meta-analysis by Li et al., the authors pooled 10 GBM studies and reported that *EGFR* amplification was associated with worse OS (pooled HR: 1.57, 95% CI 1.15–2.14), but 50% of their studies were again from before 2010.^42^ In both studies, IDH and *MGMT* status were not accounted for, and the data were mostly from the time before the wide use of TMZ. In an unselected population without known IDH status, lack of *EGFR* amplification may be a surrogate of an IDH-mutant glioma, which may lead to the observation that a lack of *EGFR* amplification is associated with better prognosis. Given our study analyzed *EGFR* amplification among 277 confirmed IDH-wildtype GBM patients with known *MGMT* status treated in the modern era, the negative finding suggests that *EGFR* amplification is likely not a meaningful prognostic biomarker for IDH-wildtype GBM. Interestingly, using 150 patients who had NGS of 315 gene panel, we observed that 87% had mutations of all 3 canonical pathways (RTK/PI3K, p53, and Rb), so *EGFR* amplification may be just one of many mechanisms for GBM tumorigenesis and thus may not carry any prognostic impact.

Given the current study is retrospective and derives from a single high-volume tertiary center, the findings should be considered hypothesis-generating and should be further validated. Given the relatively limited sample size of our mGBM cohort, a more extensive study is required for validation, ideally with patients treated with uniform chemoradiotherapy and known *MGMT* status. Regarding *CDKN2A/B* deletion, it was only evaluated for 41% of cases in this study. *CDKN2A/B* testing was routinely evaluated as part of an NGS panel for the more recent patients and should not be influenced by selection bias. However, validation from larger multi-institutional studies is warranted to confirm its prognostic impact. As mentioned earlier, given the relative rarity of *TERT*-wildtype status, our study may be underpowered to detect a small prognostic impact. However, this would also suggest that it is unlikely to have a significant impact on patient stratification as the vast majority of GBM patients will have *TERT* mutation. Our study purposely included all GBM patients who received at least 1 fraction of RT with or without TMZ to try to capture the real-world experience and to minimize selection bias. Subset analyses with patients who received SRT and TMZ also showed the same results, thus suggesting treatment heterogeneity should not affect the main findings.

In summary, *CDKN2A/B* deletion, but not *TERT* mutation nor *EGFR* amplification, appears to be an independent prognostic biomarker for IDH-wildtype GBM. Although mGBM based on the current cIMPACT-NOW criteria has relatively poor OS and PFS, its clinical outcomes may not be identical to that of hGBM after chemoradiotherapy, so further refinement and validation may be needed.

## Supplementary Material

vdaa126_suppl_Supplementary_Tables

## Notes

**Conflict of interest statement**. J.H. reports research support from Pfizer, outside the submitted work. J.L.C. reports research support from NeoImmuneTech Inc. and Incyte Corporation, outside the submitted work. G.P.D. is a cofounder of Immunovalent, outside the submitted work. A.H.K. is a consultant for Monteris Medical and received research grants from Monteris Medical, Stryker, and Collagen Matrix, outside the submitted work. The remaining authors declare no conflict of interest.

## Notes

**Authorship Statement**. Study design: S.R. and J.H. Data collection: S.M., S.R., J.L.C., S.D., G.P.D., T.J., M.G., A.H.K., and J.H. Analysis and interpretation: S.M., S.R., J.L.C., S.D., G.P.D., T.J., A.H.K., and J.H. Writing: S.M., S.R., and J.H. Manuscript review and approval: S.M., S.R., J.L.C., S.D., G.P.D., T.J., M.G., A.H.K., and J.H.
