# Supplementary material for: Prognostic impact of CDKN2A/B deletion, TERT mutation, and EGFR amplification on histological and molecular IDH-wildtype glioblastoma
Source: Neurooncol Adv. 2020 Sep 18;2(1):vdaa126. doi: 10.1093/noajnl/vdaa126 (PMC7668466; doi:10.1093/noajnl/vdaa126)
Supplement: vdaa126_suppl_Supplementary_Tables [file vdaa126_suppl_supplementary_tables.docx]

**Supplemental Table S1.** Univariable and Multivariable analysis for PFS

| **Characteristic** | **UVA** | **p-value** | **MVA** | **p-value** |
| --- | --- | --- | --- | --- |
| Age at diagnosis | 1.01 (1.00 – 1.02) | 0.01 | 1.00 (0.99 – 1.01) | 0.79 |
| KPS | 0.98 (0.97 – 0.98) | <0.001 | 0.98 (0.97 – 0.99) | <0.001 |
| Male Sex | 1.14 (0.91 – 1.43) | 0.25 | -- | -- |
| Non-white race | 1.00 (0.62 – 1.61) | 0.99 | -- | -- |
| mGBM | 0.65 (0.40 – 1.06) | 0.08 | 0.43 (0.26 – 0.72) | 0.001 |
| Extent of Resection  GTR  STR  Biopsy | Ref  1.61 (1.24 – 2.09)  2.70 (2.03 – 3.59) | <0.001  <0.001 | 1.76 (1.34 – 2.30)  2.91 (2.15 – 3.94) | <0.001  <0.001 |
| Unmethylated *MGMT* | 1.88 (1.48 – 2.40) | <0.001 | 1.97 (1.53 – 2.53) | <0.001 |
| HFRT | 1.79 (1.34 – 2.40) | <0.001 | 1.08 (0.76 – 1.55) | 0.66 |
| No TMZ chemotherapy | 2.49 (1.76 – 3.52) | <0.001 | 1.67 (1.14 – 2.44) | 0.008 |
| *TERT* mutation (n = 184) | 1.44 (0.81 – 2.56) | 0.21 | ~~--~~ | ~~--~~ |
| *EGFR* amplification by FISH or NGS (n=277) | 1.24 (0.95 – 1.61) | 0.11 | -- | -- |
| *CDKN2A/B* deletion by NGS (n=150) | 1.36 (0.93 – 1.98) | 0.11 | -- | -- |
| Pathways Affected  Three pathways  Two Pathways  One Pathway | Ref  0.69 (0.35 – 1.37)  0.56 (0.23 – 1.39) | 0.29  0.21 | -- | -- |

Abbreviations: as in Table 1.

**Supplemental Table S2:** Multivariable analysis of OS and PFS for all patients with known *TERT* mutation status (n=184)

| **Characteristic** | **OS** | **p-value** | **PFS** | **p-value** |
| --- | --- | --- | --- | --- |
| Age at diagnosis | 1.01 (0.99 – 1.03) | 0.48 | 1.01 (0.99 – 1.02) | 0.44 |
| KPS | 0.98 (0.97 – 0.99) | 0.007 | 0.98 (0.96 – 0.99) | 0.001 |
| mGBM | 0.46 (0.22 – 0.93) | 0.03 | 0.39 (0.21 – 0.74) | 0.004 |
| Extent of Resection  GTR  STR  Biopsy | Ref  2.24 (1.39 – 3.62)  3.25 (2.06 – 5.13) | 0.001  <0.001 | Ref  2.18 (1.41 – 3.37)  3.84 (2.46 – 5.98) | <0.001  <0.001 |
| Unmethylated *MGMT* | 1.56 (1.04 – 2.33) | 0.03 | 2.14 (1.46 – 3.14) | <0.001 |
| HFRT | 1.69 (0.98 – 2.90) | 0.06 | 0.94 (0.56 – 1.59) | 0.82 |
| No TMZ chemotherapy | 1.52 (0.86 – 2.70) | 0.15 | 1.25 (0.74 – 2.13) | 0.41 |
| *TERT* mutation by NGS | 0.64 (0.34 – 1.18) | 0.15 | 0.94 (0.51 – 1.75) | 0.85 |

Abbreviations: as in Table 1.

**Supplemental Table S3:** Multivariable analysis of OS and PFS for all patients with known *EGFR* amplification status (n=277)

| **Characteristic** | **OS** | **p-value** | **PFS** | **p-value** |
| --- | --- | --- | --- | --- |
| Age at diagnosis | 1.01 (0.99 – 1.02) | 0.36 | 1.00 (0.99 – 1.02) | 0.73 |
| KPS | 0.98 (0.97 – 0.99) | 0.002 | 0.98 (0.97 – 0.99) | <0.001 |
| mGBM | 0.46 (0.25 – 0.82) | 0.009 | 0.44 (0.25 – 0.75) | 0.002 |
| Extent of Resection  GTR  STR  Biopsy | Ref  1.91 (1.34 – 2.72)  3.49 (2.41 – 5.07) | <0.001  <0.001 | Ref  2.08 (1.49 – 2.89)  3.19 (2.24 – 4.55) | <0.001  <0.001 |
| Unmethylated *MGMT* | 1.74 (1.28 – 2.37) | <0.001 | 2.09 (1.55 – 2.83) | <0.001 |
| HFRT | 1.63 (1.02 – 2.61) | 0.04 | 1.14 (0.73 – 1.76) | 0.57 |
| No TMZ chemotherapy | 1.52 (0.95 – 2.44) | 0.08 | 1.45 (0.93 – 2.27) | 0.11 |
| *EGFR* amplification by FISH or NGS | 1.19 (0.90-1.58) | 0.22 | 1.26 (0.96-1.65) | 0.10 |

Abbreviations: as in Table 1.
